# Migration of Sri Lankan medical specialists

**DOI:** 10.1186/1478-4491-11-21

**Published:** 2013-05-21

**Authors:** A Pubudu De Silva, Isurujith Kongala Liyanage, S Terrance GR De Silva, Mahesha B Jayawardana, Chiranthi K Liyanage, Indika M Karunathilake

**Affiliations:** 1 Medical Services Division, Ministry of Health, Colombo, Sri Lanka; 2 Faculty of Medical Sciences, Department of Pharmacology, University of Sri Jayawardenapura, Colombo, Sri Lanka; 3 Faculty of Management, Social Sciences and Humanities, General Sir John Kotelawala Defence University, Colombo, Sri Lanka; 4 Medical Education Development and Research Unit, Faculty of Medicine, University of Colombo, Colombo, Sri Lanka

**Keywords:** Migration of health-care workers, Health-care worker shortage, Training of medical specialists

## Abstract

**Background:**

The migration of health-care workers contributes to the shortage of health-care workers in many developing countries. This paper aims to describe the migration of medical specialists from Sri Lanka and to discuss the successes and failures of strategies to retain them.

**Methods:**

This paper presents data on all trainees who have left Sri Lanka for postgraduate training through the Post Graduate Institute of Medicine, University of Colombo, from April 1980 to June 2009. In addition, confidential interviews were conducted with 30 specialists who returned following foreign training within the last 5 years and 5 specialists who opted to migrate to foreign countries.

**Results:**

From a total of 1,915 specialists who left Sri Lanka for training, 215 (11%) have not returned or have left the country without completing the specified bond period. The majority (53%) migrated to Australia. Of the specialists who left before completion of the bond period, 148 (68.8%) have settled or have started settling the bond. All participants identified foreign training as beneficial for their career. The top reasons for staying in Sri Lanka were: job security, income from private practice, proximity to family and a culturally appropriate environment. The top reasons for migration were: better quality of life, having to work in rural parts of Sri Lanka, career development and social security.

**Conclusions:**

This paper attempts to discuss the reasons for the low rates of emigration of specialists from Sri Lanka. Determining the reasons for retaining these specialists may be useful in designing health systems and postgraduate programs in developing countries with high rates of emigration of specialists.

## Introduction

Most countries face a shortage of health-care workers (HCWs), irrespective of their developmental status [1]. According to the estimates of the World Health Organization (WHO), there was a shortage of 4.3 million HCWs worldwide by the year 2006. However, this shortage does not affect countries equally; for example, there is a 27-fold difference in the number of psychiatrists per 100,000 persons between India and the United Kingdom [1]. Furthermore, there are significant differences between human resources even among countries of a similar financial status [2].

One response of those countries facing shortages is to encourage the recruitment of health-care personnel from other countries [3]. The international recruitment of HCWs has both positive and negative effects on the source country. It creates opportunities for HCWs to develop their careers, gain valuable experience and enjoy better living conditions, especially if the recipient country is more developed [4]. Their country of origin may also benefit from foreign currency remittances, transfer of experience and knowledge [5]. However, the main negative effect of migration is that it deprives the source country, which invested a significant amount of resources to educate and train these HCWs, of their services [3].

The literature on the migration of HCWs identifies factors that attract them to developed countries (pull factors) and those that deter them from remaining in the source country (push factors) [6,7]. Research has identified several prominent pull factors [6], such as, more opportunities for professional training, higher salaries and benefits and better living conditions. Those that are identified as push factors are low wages, poor or dangerous working conditions, surplus production of HCWs, unemployment, underemployment and lack of opportunities for professional development [7]. In addition, factors associated with the socio-political environment, such as ethnic inequalities, human rights violations, corruption, political persecution and economic instability are also identified as factors that encourage emigration [8].

Medical specialists are among the most sought after category of HCW across the world, including most developed countries. Countries adopt various strategies, such as providing various incentives and migration programs, to attract experienced specialists from other countries. In response, many governments in developing countries have been trying to formulate strategies to minimize the emigration of specialists. Several organizations, including the World Health Organization (WHO), have responded to this situation by publishing codes of practice for the international recruitment of HCWs [9], which are aimed at ensuring that the health-care delivery of the source country is not affected by such migration.

### Status of Sri Lanka

Sri Lanka, as a middle income country with just 4% of its gross domestic product (GDP) dedicated to health, is no exception in having a shortage of medical specialists and doctors; in 2010, the ratio was 1:1,462 [10]. In spite of this, many health-related indices provide evidence that Sri Lanka has achieved good health standards when compared to countries of similar economic status [11].

The country’s education system has attempted to respond to this shortage by increasing the intake of medical students and establishing new medical schools. The country has had a free educational system since 1945, providing education from primary to tertiary level in all state schools and universities, which includes medical degrees.

### Training and recognition of medical specialists in Sri Lanka

The Postgraduate Institute of Medicine (PGIM) of the University of Colombo is the only postgraduate institution for medical doctors in Sri Lanka. Board certification as a specialist, which is recognized by the Sri Lanka Medical Council (SLMC), can only be achieved via the PGIM training programs.

Doctors who want to qualify as a specialist in Sri Lanka need to pass a selection exam for their specialty before starting training as a trainee/registrar at PGIM. A second exam is held after basic training (varying from 2 to 4 years depending on the specialty). Trainees who pass are eligible to undergo advanced training (as senior registrars). After completing the advanced training the trainees are required to undergo a compulsory period of foreign training (1 to 2 years). After completion of foreign training the specialists will be board certified as medical consultants in the relevant fields. Overseas training placements include centers in the United Kingdom, Australia, Singapore and New Zealand.

Overseas training has contributed to an improvement in training outcomes and producing better, up-to-date specialists who meet international standards [12]. The government of Sri Lanka ensures the accessibility of postgraduate studies in medicine by making it free of charge for those who qualify. Further, it provides stipends, airfare and other incentives for specialists during their overseas training. Thus, the government invests a large amount of resources in the development of specialists.

This investment at postgraduate training level is, understandably, subject to several conditions. When accepted for training, the specialists are required to submit to a bond agreeing to return and work in Sri Lanka after their training: they must work four years for every year they spend in overseas training. If they fail to complete the bond period, they are liable to reimburse the stipend and the salary which they received during their foreign training period.

## Scope of study and methodology

There is a paucity of evidence on the extent of emigration of health-care workers from Sri Lanka. In this study we aim to assess the situation in Sri Lanka with regard to the migration of medical specialists and to discuss the successes and failures in the strategies adopted to retain them.

All postgraduate trainees who left for postgraduate training since 1980, the inception of the PGIM of the University of Colombo, up to June 2009 were included in the study. Data from official records of both the PGIM and the Ministry of Health were obtained. A sub-analysis was carried out on trainees who left from January 2006 to June 2009.

A qualitative review was carried out on specialists who have completed their training. It was a confidential, telephone-based, semi-structured interview that was carried out to inform the Ministry of Health as a quality improvement program led by the first author. A sample of specialists who have returned to Sri Lanka or emigrated was selected randomly from the PGIM registries of the last 5 years. The interviews were conducted by the researchers themselves, and anonymity and confidentiality were strictly maintained.

The selected specialists were contacted via email to obtain consent. Although all 30 specialists residing in Sri Lanka were accessible and consented to participate in the study, only 5 who have emigrated responded. The specialists who consented were interviewed using direct and open-ended questions. Specialists were also asked to list five reasons for their decision to remain in Sri Lanka or leave Sri Lanka, and specify whether the reasons are high, medium or low priority.

In addition, data was collected from existing records at the Ministry of Health and the Post Graduate Institute for Medicine (PGIM) using standard, structured, data sheets and analyzed using the SPSS 10 statistical package.

## Findings

Since the inception of the PGIM in 1980 up to June 2009, a total of 1,915 specialists have undergone foreign training and have been certified as consultants by the PGIM. By 2010 there were a total of 1,042 specialists working in the government sector while most others have retired having reached the retirement age. All the specialists had signed for a bond period of 4 years with the Ministry of Health before engaging in foreign training. Among the 1,915 specialists, 215 (11%) migrated before the completion of the bond period. The total bond value due to the government from those specialists who left without completion of the bond period is SLR 215,475,884.76 (approximately USD 1,657,507). A majority (*n* = 148, 69%) have settled the bond or started paying it off, while legal action to recover the bond is required for the remaining 67 specialists. The total amount due from these 67 postgraduate trainees/specialists is SLR 97,659,002.70 (approximately USD 751,224).

From January 2006 to June 2009 a total of 579 specialists left Sri Lanka for foreign training, of which 76 (13%) migrated before completion of the bond period. The numbers of specialists in different specialties who have undergone foreign training and those who have migrated from January 2006 to June 2009 are listed in Table 1.

**Table 1:** Numbers of specialists who have undertaken foreign training from January 2006 to June 2009

**Specialty**	**Number of specialists in Sri Lanka (by 2010)**	**Number of specialists who undertook foreign training from 2006 to 2009**	**Number (%) of specialists who migrated from 2006 to 2009**
Internal medicine and other medical specialties	281	156	23 (15)
General surgery and other surgical specialties	201	67	14 (21)
Anesthesia	82	51	10 (20)
Psychiatry	18	38	5 (13)
Other specialties	460	267	24 (9)
**Total**	**1,042**	**579**	**76 (13)**

Considering the different countries in which the trainees had trained during January 2006 to June 2009, most of the trainees had undergone training in Australia and the United Kingdom (Table 2). Among those who migrated after training from January 2006 to June 2009, 52.6% migrated to Australia while 38.2% migrated to the United Kingdom.

**Table 2:** Number of specialists by country where training was undertaken from January 2006 to June 2009

**Country**	**Number of trainees who have undertaken training from 2006 to 2009**	**Number of trainees (%) who have migrated or not returned from 2006 to 2009**
Australia	252	40 (16)
United Kingdom	202	29 (14)
New Zealand	45	7 (16)
Singapore	28	0
Other countries	52	0
**Total**	**579**	**76 (13)**

### Analysis of feedback

Specialists who returned to Sri Lanka after their training acknowledged that foreign training provided them with an opportunity to improve their knowledge and skills. Identified areas of importance in foreign training are: clinical skills, communication skills, patient-centered care, management of chronic conditions, geriatric care, advanced surgical training and methods, teamwork and the multidisciplinary care and research culture. They felt that foreign training is an essential component in the development of a specialist, and that it should be continued to ensure the quality of specialists meets international standards.

In addition, 16 of the specialists (53%) who returned to Sri Lanka stated that foreign training was an opportunity to strengthen their financial status as they were able to save money during their training period.

When asked about the reasons for their decision to stay in Sri Lanka, the participants identified the following reasons. The need to live in the same country as their extended family was identified by 29 out of 30 (97%) participants. They also mentioned the need to support their siblings and provide care for their parents. Among other reasons, 24 specialists (80%) believe that Sri Lanka is more suitable for their children, as it is more ‘culturally appropriate’, with ‘better schooling choices’ and family support in raising children. Another reason was their wish to serve their country and its people, who paid for their education.

Sixteen specialists (53%) believed that Sri Lanka is financially more beneficial than the country in which they were trained. It was understood that they were referring to the income from private practice in Sri Lanka.^a^

Job security and stability were listed as incentives for remaining in Sri Lanka, identified by 18 (60%) and 16 (53%) of the participants, respectively. Further, as clarification, they had taken into consideration the permanent status of their employment where further placements in better positions will automatically become available as they become more senior.

Of the specialists who migrated from Sri Lanka, four cited the compulsory appointment to a rural area once they return as the main reason for migration. This was prompted by their view of the standard of schools and other facilities in these rural areas, which they felt would reduce their quality of life significantly. In addition, all five specialists who had migrated shared the following as reasons for their decisions to migrate: better working hours, better quality of life, better education for their children, better social security and a better working environment. They also mentioned the lack of professional development and opportunities to develop their career once they become specialists in Sri Lanka. Only two of the five specialists stated that they were considering returning to Sri Lanka later in their lives.

## Discussion

Sri Lanka has been able to maintain emigration rates for medical doctors at a lower level than many developing countries [13,14]. Australia, the United Kingdom and New Zealand are the countries to where most of the specialists have migrated. During the time period of this study, all specialists who trained in Singapore have returned.

A higher salary has been identified as an important factor in retaining health-care workers in a country [15]. This study however, showed mixed responses from specialists with regard to the financial benefits of migrating to a developed country. Almost half of the specialists who returned to Sri Lanka believed it is more beneficial to remain in Sri Lanka financially. In contrast, all five specialists who emigrated believed they are more socially secure and the quality of life is better when living in a developed country.

The difference in the salary of a medical specialist in a developed country and a developing country is extensive. A physician in Australia is paid approximately 30 times more than a similarly qualified counterpart in Sri Lanka [16]. Vujicic et al. suggested that a slight increase in the wages of specialists in a developing or underdeveloped country may not have a significant effect on this gap, and thus may not significantly contribute in retaining the specialists [17]. They suggested that other non-wage concessions and remuneration may be more effective in minimizing the emigration of health-care workers.

Currently, the government provides many incentives to skilled workers in Sri Lanka. These include priority in school admissions, duty concessions for importing a vehicle for personal use, a pension after retirement and other financial remunerations, such as loans at low interest. In addition, Sri Lankan society recognizes the medical profession as a one of the most important professions. This recognition, respect and prominence in society may be important factors in discouraging emigration.

According to Sri Lankan legislation, medical specialists and doctors are allowed to work in the private sector after duty hours. There are no limitations on work hours or the number of patients that can be seen in the private sector. A majority of the medical specialists in Sri Lanka, practice in the private sector after duty hours (before 8am and after 4pm). The earnings from working in the private sector while continuing to work in the government sector provide a specialist with many times the salary of the government sector, although it involves them working several hours extra. The fact that some specialists preferred rural areas because there is less competition for private patients may be highly advantageous in providing specialists for low-resource areas.

This has created a unique, self-funded system where the specialists receive a good income that is comparable to what they would receive in the developed world while they remain in their own country. This has also reduced the workload in state hospitals, especially in the provision of outpatient care. Currently approximately 50% of outpatient care in Sri Lanka is provided by the private sector [18]. This combined system of practice has also made specialists more accessible to the general public with minimal waiting times for consulting a specialist.

However, there are drawbacks in allowing doctors to work in both the public and private sectors. Dual practice is known to create conflicts of interest among doctors and reduce the quality of care delivered in the public sector [19]. Therefore it is important to achieve a balance between the quality of care and the number of patients seen. How private practice affects the performance of physicians and the quality of care they provide in this setting needs further evaluation and it is a potential area for further study.

During the war, many were reluctant to work in war-torn areas due to safety reasons. With the end of the 30-year war against terrorism, migration would be further discouraged through improvements in the road network and other modes of transport and economic development.

The postgraduate training program of the PGIM may also contribute to the low levels of specialist emigration. Initial training (basic and advanced) is carried out in Sri Lanka before the trainees are sent for overseas training. This exposes the trainees to the environments, disease profiles and patient populations of the local and developed setting. It can be concluded that the specialists who remain in Sri Lanka do so while being more informed of the choices they have. This model of training may be useful for developing countries that plan to establish postgraduate training programs of their own.

This survey was conducted with the Ministry of Health, Sri Lanka. This may have led to biased responses by some of the respondents. An additional drawback of this study was the low number of emigrant specialists who participated. The study group tried to contact 16 specialists and was only able to interview 5. These difficulties in involving workers who have emigrated are identified as an obstacle in carrying out studies on this issue [15,20]. Further, this study did not include specialists who resigned or migrated after completion of the bond period.

## Conclusion

The retention rate of specialists within the bond period after training was 87% in Sri Lanka. Job security, social recognition and income generated from private practice may have contributed to the low rates of emigration compared to similar countries. The postgraduate training program with training both locally and overseas has been successful in creating qualified specialists who are willing to remain in Sri Lanka. These strategies may be useful for other developing countries in planning their health systems and postgraduate training programs to reduce the emigration of medical specialists.

## Endnotes

^a^All the clinical specialists in this study were involved in private practice in Sri Lanka.

## Abbreviations

HCW: Health-care worker; PGIM: Postgraduate Institute of Medicine; WHO: World Health Organization.

## Competing interest

The authors declare that they have no competing interests.

## Authors’ contributions

APDS, IKL and STGRDS were involved at all stages of the study from the stage of planning. MBJ and CKL were involved in analysis and writing the article and editorial work. IMK supervised the project. All authors read and approved the final manuscript.
